# Using Real World Evidence to Describe Pulmonary Arterial Hypertension Treatment Patterns, Healthcare Resource Utilization, and Costs Associated with PDE-5 Inhibitor Monotherapy

**DOI:** 10.36469/9812

**Published:** 2018-01-09

**Authors:** George Ruiz, Jason Yeaw, Cassandra A. Lickert, Ajita P. De, Rolin L. Wade, Janis Pruett, William Drake

**Affiliations:** 1 Pulmonary Hypertension Program Medstar Heart Institute, Washington, DC, USA; 2 IQVIA, Plymouth Meeting, PA, USA; 3 Actelion Pharmaceuticals US, Inc., South San Francisco, CA, USA

**Keywords:** real world evidence, tadalafil, sildenafil, pulmonary arterial hypertension, healthcare utilization

## Abstract

**Background:** Pulmonary arterial hypertension (PAH) is described by proliferation of small pulmonary arteries leading to increased pulmonary vascular resistance, right ventricular failure, and death. Research confirms long-term improvement in composite morbidity and mortality endpoints on some endothelin receptor antagonists alone and in combination with phosphodiesterase type 5 inhibitors (PDE-5is) but not with PDE-5i monotherapy. While current treatment guidelines incorporate these findings, a substantial number of patients are started or maintained on PDE-5i monotherapy.

**Objectives:** This study describes real-world clinical practice and treatment patterns with PDE-5i monotherapy including events indicative of clinical worsening, treatment modifications, adherence, allcause healthcare resource utilization, and costs.

**Methods:** This retrospective study analyzed PharMetrics Plus claims data including 150 million lives; study period was January 1, 2009 through December 31, 2013. Eligible patients were ≥18 years with ≥1 inpatient or ≥2 outpatient claims ≥30 days apart, a diagnosis of pulmonary hypertension or other chronic pulmonary heart disease, and an initial PDE-5i prescription. To include only World Health Organization group 1 PAH patients, ≥1 encounter for right-heart catheterization or Doppler echocardiogram was required during the pre-index period.

**Results:** PDE-5i monotherapy for PAH treatment was associated with high treatment modification rates, low adherence, increased healthcare resource utilization, and high costs. At 12 months post index, 41.5% of patients experienced treatment modification. For the index therapy, 47% of patients had ≥80% adherence to therapy. Almost 50% of patients had ≥1 hospitalization, with costs increased three fold to $197 111 compared to $59 164 for non-hospitalized patients.

**Conclusions:** Initial treatment with PDE-5i monotherapy was associated with substantial direct medical costs, including hospitalizations and emergency department visits, low therapy adherence and a high rate of treatment modifications.

## BACKGROUND

Pulmonary arterial hypertension (PAH) is characterized by proliferative vasculopathy of the small pulmonary arteries leading to increased pulmonary vascular resistance and eventual right ventricular failure and death.[1] It is a rare disease with an annual United States (US) incidence of 2.3 and prevalence of 12.4 cases/million.[2] Recent estimates from the Registry to Evaluate Early and Long-term Pulmonary Arterial Hypertension Disease Management (REVEAL) indicate median survival of >7 years following diagnosis for patients receiving PAH specific treatment, with a 15% 1-year incident mortality rate.[3]

Substantial therapeutic progress has been made over the past 20 years. Currently there are five approved medication classes: endothelin receptor antagonists (ERAs), phosphodiesterase type 5 inhibitors (PDE-5is), guanylate cyclase stimulators, prostacyclin analogues, and a selective prostacyclin (IP) receptor agonist. Effective as monotherapies, there is also compelling evidence that initial or early treatment with combination regimens improves progression-free survival (PFS), indicated by slowing disease progression and reducing hospitalizations.[4-6] Results from several long-term trials[4-6] confirm improvements in PFS with ambrisentan in combination with tadalafil, macitentan and selexipag alone, or in combination with other agents. No evidence exists for ambrisentan monotherapy or commonly prescribed PDE-5i monotherapy.

The most recent European Society of Cardiology/European Respiratory Society (ESC/ERS) guidelines[7] provide a risk-based assessment strategy to guide PAH treatment. "Patients can be classified as low, intermediate or high risk for clinical worsening or death", with an overall treatment goal of achieving low risk status with (characterized by good exercise capacity, favorable quality of life, and good right ventricular function) <5% 1-year mortality.[7] Patients at intermediate risk may start with monotherapy or combination therapy. Monotherapy agents include PDE-5is, ERAs, guanylate cyclase stimulators or an IP receptor agonist. High-risk patients should begin with initial double or triple combination therapy including an intravenous prostacyclin analogue.

Despite risk-based guidelines, diagnostic advances, and availability of efficacious medical therapies, PAH remains an incurable, progressive disease with persistent gaps in care, delays in both diagnosis and initiation of treatment, and inappropriate medical management.[8-12] PAH imposes significant economic burden on patients and US payers, with high costs associated with pharmacy, inpatient, and outpatient services.[11,13-19] As a rare disease, research data from randomized clinical trials (RCTs) are limited. Healthcare decision makers, charged with coverage decisions and payment policies, increasingly seek information on real world (RW) outcomes.[20]

Real world evidence (RWE) challenges the traditional view that only medical evidence generated through RCTs can be incorporated into clinical practice. RWE taps into large volumes of data, and weaves together data sources to yield an enhanced picture of individual patient characteristics and improve the ability to address individual patient needs.[21] These data can be derived from a number of sources: health surveys, registries, electronic medical records, pragmatic trials and claims databases. Large claims database research provides healthcare decision makers and sponsors with additional data to address unanswered clinical practice issues in RCTs. The cohort of patients in RCTs is necessarily limited to a homogenous population under controlled conditions. In PAH, trials are generally shorter and smaller and may not address the heterogeneous population seen in clinical practice. RWE goes beyond traditional RCTs to provide:

1. Outcomes for complex or co-morbid patients not usually included in RCTs.
2. Outcomes demonstrating impact in PAH sub-populations (ie, Portal Pulmonary Hypertension, HIV patients).
3. Economic impact of product use, (ie, overall medical costs or utilization of medical services).

Real-world data, however, have limitations. Claims databases are not complete patient health records. Data are collected to carry out the daily practice of medicine, for billing and other business purposes, but are not specifically designed for patient research and may have some gaps. For example, in PAH, claims data cannot provide patient functional class (FC).

RWE does have a place in healthcare; it is essential for sound coverage and reimbursement decisions.[20] Even before the moniker, "real world evidence" existed, findings from observational studies were crucial in supporting clinical interventions. For example, in the 1990s a Medicare sponsored initiative, the Cooperative Cardiovascular Project, utilized over 200 000 records from patients with Myocardial Infarcts (MI) to evaluate the use of beta blockers and the effects on mortality.[21,22] This evidence augmented previous clinical trial data and helped to support a new practice standard—use of beta blockers in MI patients.[21,22]

Over the last several years, a number of real world evidence studies have added to the body of knowledge in PAH.[14,23] A retrospective database study showed PAH (FC II-IV) patients had high healthcare resource utilization with annual pharmacy costs 17 to 21 times higher and medical costs 10 to 11 times higher than the average Medicare patient.[24] A prospective analysis of newly diagnosed PAH patients from REVEAL showed a substantial burden of disease with a 57% incidence of all-cause hospitalization (52% PAHrelated) and worse survival at 3 years.[25] Those charged with making managed care formulary decisions which optimize the care of patients and manage resources, are searching for ways to offset high costs. Step edits and limited formularies are among the tools used to attempt to improve the cost effectiveness of therapeutic selections.[26]

Understanding of the burden of PAH and current management practices are essential to improve patient outcomes and inform decisions makers. The primary objective of this study was to identify and describe real-world clinical practice and treatment patterns including demographic, clinical characteristics, shortterm clinical outcomes, treatment modifications (Appendix 1) and adherence of patients newly initiating PDE-5i monotherapy. A secondary objective was to characterize all-cause healthcare resource utilization and costs among these patients prior to treatment discontinuation, medical therapy switch, or treatment augmentation.

## METHODS

### Study Design

This retrospective cohort study utilized the nationally representative PharMetrics Plus Healthcare claims database, with > 150 million unique enrollees in 50 states during the analysis. The database contains mainly commercially insured (commercial and self-insured, which is a subset of commercial plans) (94.7%), Medicare Advantage Prescription Drug (3.5%), Medicaid (1.5%), and unknown (0.5%) with pharmacy and medical claims (Table 1). Patient demographics, health plan and payer type(s), provider type(s), ICD-9-CM diagnoses, Charlson Comorbidity Index (CCI),[27] and healthcare resource utilization is also available.

### Patient Selection

Patient selection period was January 1, 2009 through December 31, 2013 (Figure 1) with first claim for a PDE-5i defined as the index date. A right-heart catheterization (RHC) or Doppler echocardiogram was required during the 180 day pre-index period. Continuous plan enrollment was required for 6 months preindex and 12 months post-index.

**Figure 1.** Study Design and Pre- and Post-index Periods Including Patient Selection Window for Index Event

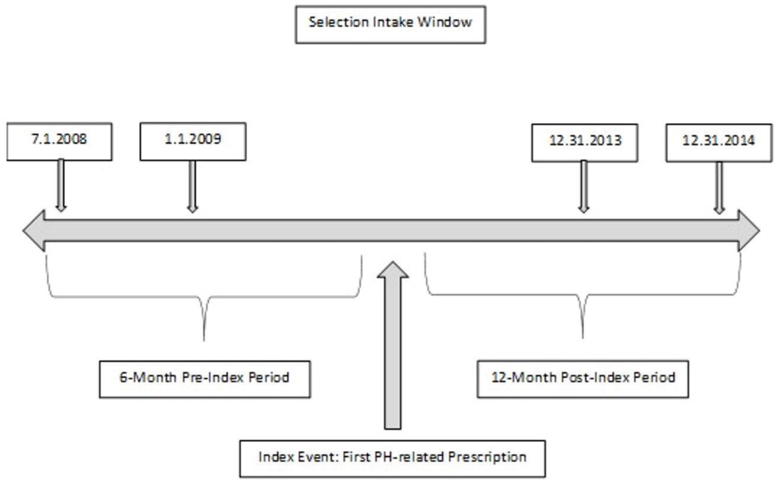

PH: pulmonary hypertension

Eligible patients were ≥18 years at index date with ≥1 inpatient or ≥2 outpatient claims at least 30 days apart with a diagnosis code for pulmonary hypertension (PH) (416.0) or other chronic pulmonary heart disease (416.8) and a PDE-5i prescription (sildenafil citrate [Revatio®], generic sildenafil, or tadalafil [Adcirca®]). Patients were required to have ≥1 inpatient or ≥2 outpatient diagnostic claims of pulmonary hypertension (416.0, 416.8) at least 30 days apart, ≥ 1 medical claim for a right heart catheterization or Doppler echocardiogram, and be aged 18 years or older during the 6 months on or before the date of the first PDE-5i pharmacy claim (Index date). The dose frequency for tadalafil was 40 mg/day and sildenafil was 20 mg three times a day. Patients who were using PDE-5is for purposes other than PAH, including those with a prescription claim during the pre-index period for branded Viagra® (sildenafil) or Cialis® (tadalafil) were excluded. The specific PAH therapy helps to identify PAH from the PH patients. This specific algorithm was validated in a previous study.[28]

### Statistical Analysis

Descriptive statistics were calculated with percentages for categorical variables and mean, standard deviation, and median for continuous variables. Kaplan-Meier analysis using the log-rank test evaluated differences in persistence with the index prescription and time to first hospitalization. Calculation of persistence was based on the number of consecutive days from index therapy to discontinuation, medication switch, augmentation, or the end of available data. Adherence was measured using the proportion of days covered (PDC) metric defined by Pharmacy Quality Alliance[29] and calculated as a continuous variable. The PDC measures adherence based on fill dates and days supply for each prescription.[29] The PDC was also calculated as a point estimate in quintiles (intervals of 20%), with ≥80% PDC meeting the criterion for adherence.

## RESULTS

### Patient Disposition and Characteristics

Between January 1, 2009 and December 31, 2013, there were 1465 eligible patients initially prescribed a PDE-5i medication (Table 1, Appendix figure). Index prescriptions were tadalafil (27.6%) and sildenafil (72.4%). Patients were predominantly female (59.7%) with a mean age of 58.1 (±13.0) years. The most common comorbidities were systemic hypertension (72.8%), diabetes (32.5%), thyroid disease (21.5%) and obesity (19.1%). Average overall pre index interval healthcare costs were $73 748 (±$136 227). Of all patients, 94.7%

were commercially insured (Table 1). For analysis purposes, the aggregate of all payers' types was used.

**Table 1.** Baseline Patient Demographic and Clinical Characteristics (N = 1 465)

| Characteristics | n/Mean | %/SD |
|---|---|---|
| Age (years) (mean, SD) | 58.1 | 13.0 |
| **Age (years) (n, %)** | | |
| 18-34 | 72 | 4.9 |
| 35-44 | 122 | 8.3 |
| 45-54 | 344 | 23.5 |
| 55-64 | 518 | 35.4 |
| ≥ 65 | 409 | 27.9 |
| **Gender (n, %)** | | |
| Female | 875 | 59.7 |
| Male | 590 | 40.3 |
| **PH-related therapy (n, %)** | | |
| Anticoagulants or heparins | 517 | 35.3 |
| Calcium Channel Blockers | 492 | 33.6 |
| Digoxin | 223 | 15.2 |
| Diuretics | 966 | 65.9 |
| Charlson Comorbidity Index score (mean, SD) | 3.6 | 2.2 |
| **Charlson Comorbidity Index score (n, %)** | | |
| 1 | 13 | 1.0 |
| 2 | 181 | 12.4 |
| 3 | 635 | 43.3 |
| ≥ 4 | 636 | 43.4 |
| **Comorbidities, n (%)** | | |
| Systemic hypertension | 1,067 | 72.8 |
| Diabetes | 476 | 32.5 |
| Thyroid disease | 315 | 21.5 |
| Obesity | 280 | 19.1 |
| Connective tissue disease | 256 | 17.5 |
| Liver disease | 211 | 14.4 |
| Congenital heart disease | 171 | 11.7 |
| Systemic sclerosis | 125 | 8.5 |
| Systemic lupus erythematous | 67 | 4.6 |
| Portal hypertension | 64 | 4.4 |
| Health care costs, $ | 73 748 | 136 227 |
| **Payer type, (n, %)** | | |
| Commercial | 866 | 59.1 |
| Medicaid | 22 | 1.5 |
| Medicare Advantage Prescription Drug | 51 | 3.5 |
| Self-insured* | 522 | 35.6 |
| Unknown | 4 | 0.3 |

SD: standard deviation; PH: pulmonary hypertension; n: number of patients; %: percentage *Self-insured plans are a subset of Commercial plans where the employer assumes the risk of insuring the population. All costs are pre-index costs.

### Treatment Dosing and Modifications to Index Therapy

Post-index, the overall mean gap between prescription fills was 32.5 (±36.1) days, with dose modifications summarized in Table 2. For the overall study population treatment modification was 41.5% at 12 months post index (Figure 2).

**Table 2.** Dose and Dose Modifications During Post-index Period

| MEDICATION DOSING MEASURE | PATIENTS (N=1465) |
|---|---|
| **GAP BETWEEN PRESCRIPTION REFILLS, DAYS** | |
| Mean (SD) | 32.5 (36.1) |
| Median | 21.0 |
| **DOSE MODIFICATIONS** | |
| Dose increase ≥20% among patients with ≥3 index prescriptions, n (%) | 193 (13.17) |
| Time from index date to first dose increase, days | |
| Mean (SD) | 116.0 (98.6) |
| Median | 88.0 |
| Dose decrease ≥20% among patients with ≥3 index prescriptions, n (%) | 63 (4.3) |
| Time from index date to first dose decrease | |
| Mean (SD) | 134.7 (132.2) |
| Median | 91.0 |

SD: standard deviation.

**Figure 2.** Kaplan-Meier Analysis: Time to Treatment Modification 12 Month Post-index Date
Kaplan-Meier analysis of time to treatment discontinuation, medication switch, treatment augmentation or end of study period during the 12-month post-index interval

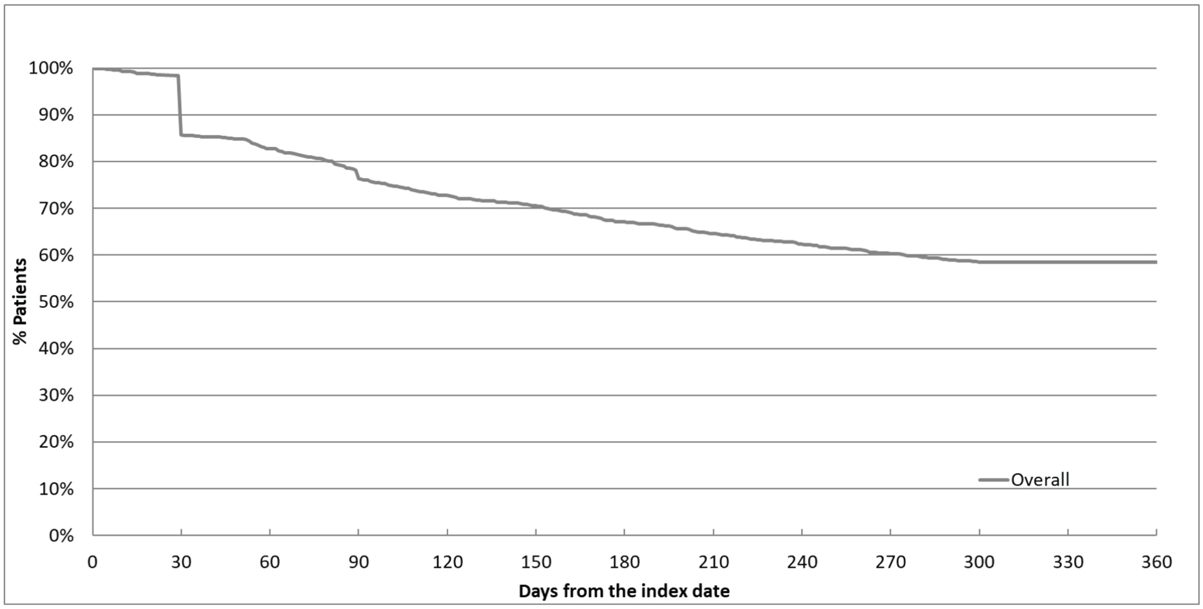

Days from index date: Index date represents the date of first PAH medication prescription.

## Adherence

For overall adherence (mean PDC of 64.1% [±31.8]), 47.0% of patients had ≥80% PDC to their index therapy. Among patients with ≥2 prescriptions, the mean overall adherence was 71.7% (±26.0), with a PDC ≥80% for 53.6% of these patients (Figure 3).

**Figure 3.** Treatment Adherence During the 12-month Post-index Period

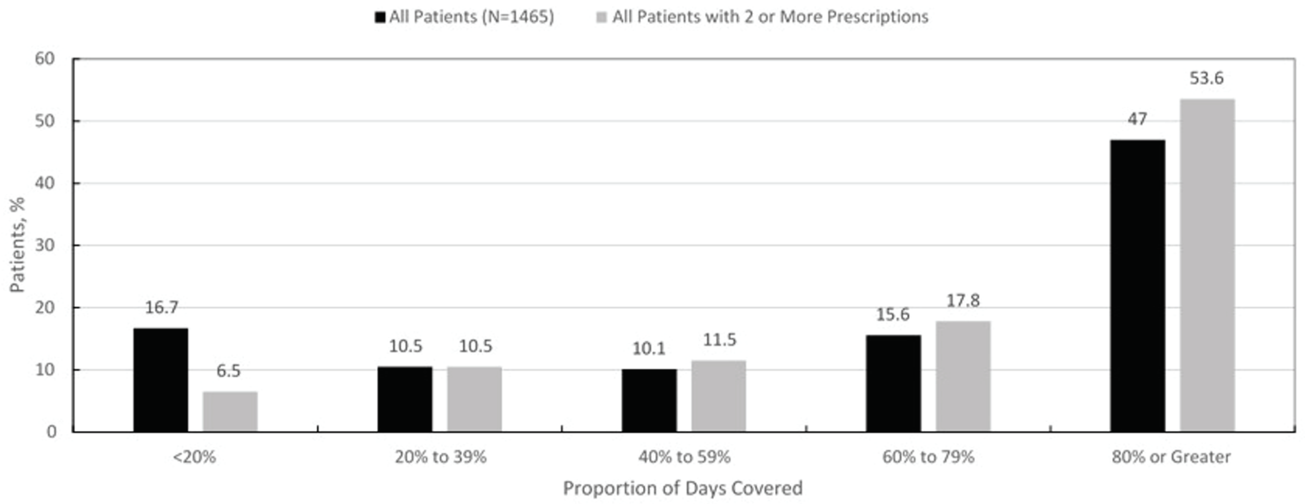

## First Hospitalization for PAH

Utilization of inpatient medical services during the post-index interval revealed total number of all-cause hospitalizations was 1455 (first admission and readmissions) and ≥1 hospital admission for 44.4% (650) of patients (Table 3). For patients with ≥1 all-cause admission (i.e., all patients that were hospitalized), mean length of stay (LOS) per hospitalization was 8.2 (±12.4) days, and mean number of admissions was 2.2 (±1.9) (Table 3). For the overall study population, the mean was 1.0 (±1.7) admission. The probability of first PAH related hospitalization was 28.2% for the overall population (Figure 4).

**Figure 4.** Kaplan-Meier Analysis: Time to First PAH-related Hospitalization During the 12-month Postindex Period

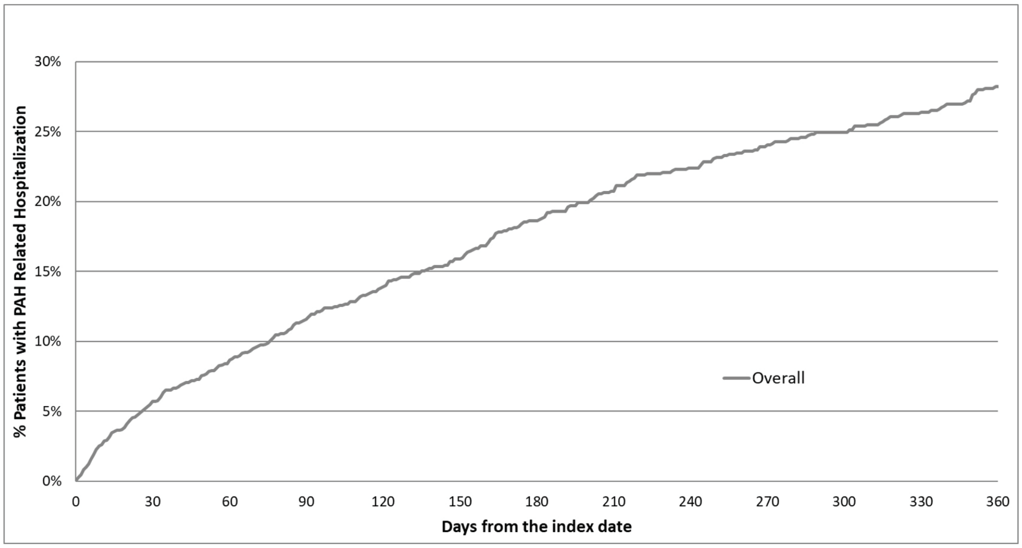

### Healthcare Resource Utilization and Costs

Claims for emergency department (ED) visits were identified for 45.3% (663 patients). For those with an ED visit, mean number of visits was 2.2 (SD ±2.2 visits) (Table 3). Pharmacy costs were comparable between hospitalized and non-hospitalized patients (Figure 5). However, mean overall healthcare costs totaled $120 369 ($189 365) rising to $197 111 ($251 651) for patients with ≥1 hospital admission compared to $59 164 ($74 488) for those not hospitalized (Figure 5).

**Table 3.** All-cause Utilization of Healthcare Resources During 12-month Post-index Interval

| HEALTHCARE SERVICE | PATIENTS (N=1465) |
|---|---|
| **OUTPATIENT SERVICES** | |
| **PHYSICIAN OFFICE VISITS** | |
| Visits for all patients, mean (SD); median 2 | 7.2 (31.9); 20 |
| Patients with ≥1 visit, n (%) | 1462 (99.8) |
| Visits among patients with ≥1 visit, mean (SD); median | 27.2 (31.9); 20 |
| **EMERGENCY DEPARTMENT VISITS** | |
| Visits for all patients, mean (SD); median | 1.0 (1.9); 0 |
| Patients with ≥1 visit, n (%) | 663 (45.3) |
| Visits among patients with ≥1 visit, mean (SD); median | 2.2 (2.2); 1.0 |
| **INPATIENT SERVICES** | |
| **HOSPITAL ADMISSIONS** | 1455 |
| Admissions for all patients, mean (SD); median | 1.0 (1.7); 0 |
| Patients with ≥1 admission, n (%) | 650 (44.4) |
| Admissions among patients with ≥1 inpatient stay, mean (SD); median | 2.2 (1.9); 2 |
| Days in hospital among patients with ≥1 inpatient stay, mean (SD); median | 20.4 (34.9); 9 |
| LOS among patients ≥1 inpatient stay, mean (SD); median | 8.2 (12.4); 5 |

LOS: length of stay; SD: standard deviation

Figure 5. Healthcare Costs Overall and for Patients with/without at Least One Hospital Admission Post-index

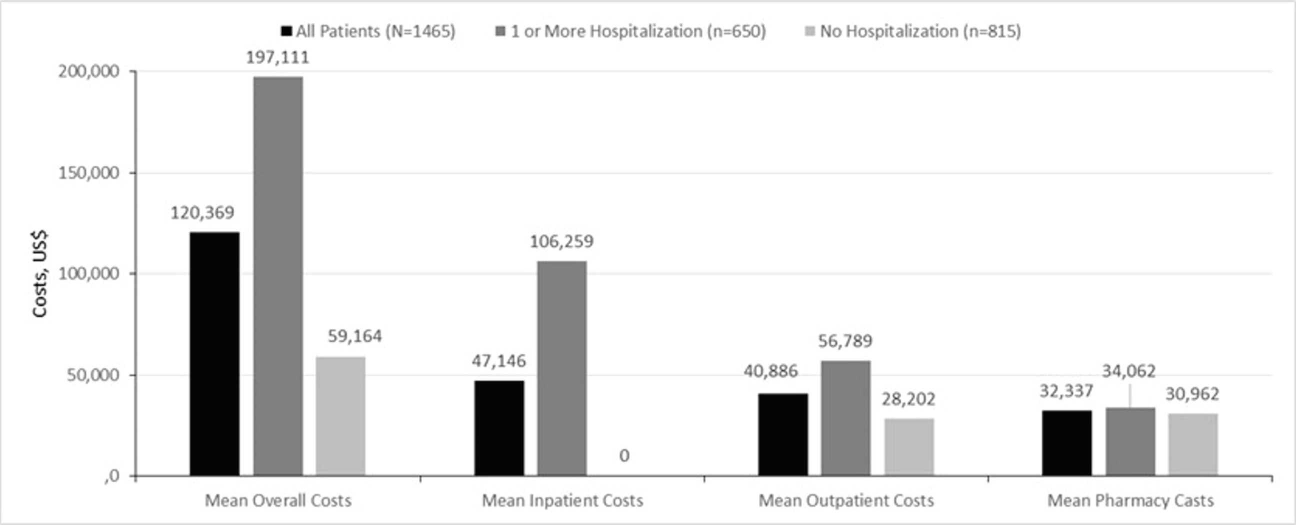

## DISCUSSION

In this real world analysis, the cohort was largely female with a mean age of 58.1 years. The most common comorbidities were systemic hypertension, diabetes, thyroid disease, obesity and connective tissue disease, consistent with those reported in the REVEAL Registry.[3] Index therapy with PDE-5i monotherapy was associated with a high rate of treatment modification including dose changes, low adherence, high healthcare resource utilization, and substantial medical costs. First-treatment modification was seen as early as 30 days post initiation. Surprisingly, nearly half of patients experienced ≥1 hospitalization with a mean of 2.2 (2.2) admissions during the 12-month follow-up period. With these hospitalizations, costs for patients with hospitalizations were approximately three times higher ($197 111) (interquartile range [IQR] 158 276) compared to costs ($59 164) (IQR 41 829) for non-hospitalized patients. Almost half of patients had at least 1 ED visit, which added additional burden to the healthcare costs. Previous claims data showed an incidence of all-cause hospitalization of 57%.[25]

The economic burden of PAH has been demonstrated in healthcare resource utilization (ER visits, outpatient visits, pharmacy fills) costs, hospitalizations (PAH related and all-cause), rehospitalization and LOS shown in both newly diagnosed and prevalent PAH patients. Several publications have estimated all-cause healthcare costs with a variable mean range of $2023 per patient per month (PPPM)17 to $9295 PPPM.[30-32] One claims analysis for adults with PAH revealed a mean of $6 617 PPPM for PAH-related expenditures, with PAH costs accounting for 71.2% of all-cause expenditures. Hospitalizations and outpatient services resulted in the highest PPPM costs.[32] Underscoring the importance of hospitalization as a cost driver, a retrospective analysis of 4009 adults with evidence of PAH, found that 56.7% experienced ≥1 PH-related hospitalization in the 3 year follow-up period.[14] Of those hospitalized, 52.9% of patients had 1 or more readmissions during the total follow-up period. Of those patients rehospitalized, 79.3% were readmitted within the first year with 23.6% having 3 or more readmissions during the first year after discharge from the initial admission.[14] The mean cost for commercially insured was $61 992 (±$213 596), with mean LOS of 14.2 (±32.3) days.[14] Similarly, in another retrospective study using a large US claims database, healthcare costs for PAH patients before and during the 12 months following initiation of PAH-indicated medication(s) revealed a significant increase in pharmacy costs from $6440 at baseline to $38 514 at follow-up.[19] Overall medical costs were lower for the follow-up interval at $59 729 compared to $110 241 before treatment initiation. Cost savings were attributed to fewer outpatient visits and hospitalizations.[19] The present study findings are consistent with recent research documenting the economic burden of PAH, which is largely driven by hospitalization.

A primary therapeutic goal for patients with PAH is to delay disease progression. Disease progression may be indicated by hospitalization, and/or switching or augmenting therapy. Previous guidelines recommended initial monotherapy with FDA approved PAH drugs, followed by regular reassessments, and combination therapy when treatment goals aimed at delaying disease progression and maintaining or achieving FC I or II are unmet.[1,33,34] An important goal in the most recent treatment guidelines (ESC/ERS),[7] is improvement to a low risk status, reflected in part by decreased hospitalization. In this study, disease progression events suggested that patients might achieve low risk status with another agent or agents. RCTs demonstrate improved morbidity/mortality events and/or hospitalizations in patients treated with macitentan and selexipag alone or in combination.[4-6] For previously untreated patients treated with combination ambrisentan and tadalafil, hospitalization and clinical failure (measured at 6 months) was reduced.[4] There is no such evidence for PDE-5i or ambrisentan monotherapy.[4] The current real-world findings indicate that initial treatment with PDE-5i monotherapy may result in greater likelihood of treatment modifications, hospitalization and economic burden.

While the ESC/ERS treatment guidelines emphasize appropriate risk assessment and risk-based treatment with monotherapy or combination therapy using medications targeting different pathways,7 patients remain at risk for therapy not consistent with evidence based guidelines.[35] For example, the Referral of Patients with Pulmonary Hypertension Diagnoses to Tertiary Pulmonary Hypertension Centers (RePHerral) study reported 57% of patients were prescribed PAH-specific medications inconsistent with guidelines.[9] Similarly, REVEAL found PDE-5i monotherapy (indicated in FC II-III only) was prescribed for 27.4% of patients who were FC III or IV.[36] For high-risk patients (FC IV), monotherapy is not deemed appropriate. The ESC/ERS guidelines strongly recommend initial double or triple combination treatment including mandatory intravenous prostacyclin analogue.[7] The recent event-driven, morbidity and mortality trials, SERAPHIN, AMBITION, and GRIPHON,[4-6] demonstrated the benefit of combination therapy on improved PFS, both as sequential or initial therapy. Additionally, in a recent meta-analysis, combination therapy demonstrated significant reduction in clinical worsening compared with monotherapy.[37] In the present study, risk status or FC could not be ascertained, thus it is not clear if the current treatment strategy is consistent with the recommended guidelines.

Currently, in an effort to control costs, many healthcare organizations are using management tools such as step edits and limited formularies.[38] Therapy choices may be based less on clinical evidence and guidelines, and more on insurance considerations, local expertise, practice patterns, and patient preferences.[39] For example PDE-5is are available in less expensive, generic formulations, therefore, health plans may implement step edits,[26] requiring patients to try generic PDE-5i agents prior to approval and reimbursement for other PAH medications. In the present study, results demonstrated that both generic and branded PDE-5is were associated with early treatment modification and significant healthcare resource utilization. The less expensive choice was not less expensive but actually more costly. In a progressive disease state such as PAH, the step therapy approach may impede clinical decisions and delay appropriate patient management.

A multitude of treatment options now exists with 13 PAH specific medications available. With these options, assessment of risk and response to therapy is critical for optimal long-term management. For lowand intermediate-risk patients, recent event-driven long-term clinical trials (SERAPHIN and GRIPHON) provide solid evidence that macitentan and selexipag, used as monotherapy delay disease progression and reduce hospitalization.[5,6] Additionally, SERAPHIN, GRIPHON, and AMBITION,[4-6] have demonstrated improved PFS using sequential or initial combination therapy.

This evidence along with appropriate assessment of which low or intermediate risk patients may benefit from monotherapy or combination therapy is important to tailor patient therapy. Using real-world research to provide evidence on patients in actual practice settings may result in cost reductions as clinicians determine when combination therapy—initial or sequential—is required, eliminating unnecessary initial combination therapy in low risk patients and potential costs associated with additional monitoring and/or management of adverse reactions. Additional real world research, including claims databases, comparative effectiveness studies, and pragmatic trials are needed to provide prescribers and patients with data for optimal PAH treatment regimens and to assist payers in making value based formulary decisions.

### Study Limitations

Several limitations must be acknowledged when interpreting these results. The study results are subject to confounding because differences in baseline characteristics across treatment groups were not controlled. No ICD-9-CM codes exist that specifically identify patients with World Health Organization (WHO) group 1 PAH in claims data.[40] Eligible patients were identified based on an algorithm similar to other PAH claims analyses,[14,24] which might have misclassified the sample.

Claims data are collected for the purpose of payment rather than to support research objectives and may be subject to coding errors. In addition, disease severity as measured by FC cannot be determined from claims data. Finally, this was an observational study and does not allow conclusions about causal relationships.

## CONCLUSIONS

Initiation of therapy for PAH with PDE-5i monotherapy was associated with substantial direct medical costs, including hospitalizations and ED visits, and a high rate of treatment modifications and low adherence to therapy. These disease progression events suggest that treatment with another agent or more than one agent may be beneficial. This study demonstrates that the choice of less expensive PDE-5is did not lead to a decrease in overall healthcare resource utilization. Thus, the economic burden of PAH (increased healthcare resource utilization and higher costs) may be exacerbated by the implementation of health plan management tools that are inconsistent with both current guidelines and evidence-based medicine (RCT and RWE). Additional research on patterns of care and costs may help identify the most effective treatment regimens for PAH patients based on specific patient risk, rather than on lower upfront cost. Such research may also help to identify patients who may obtain greatest clinical benefit from medications that demonstrate efficacy in long-term event-driven trials in monotherapy and combination therapy regimens.

## FINANCIAL/NONFINANCIAL DISCLOSURES/CONFLICTS OF INTEREST

George Ruiz is a member of speakers bureaus for Actelion Pharmaceuticals US, Inc, and United Therapeutics. Jason Yeaw, Ajita P. De and Rolin L. Wade are employees of IQVIA. Cassandra A. Lickert, Janis Pruett and William Drake are employees and shareholders of Actelion Pharmaceuticals US, Inc.

## AUTHOR CONTRIBUTIONS

JY, GR, CAL, APD, RLW, JP, WD had full access to all of the data in the study and take responsibility for the integrity of the data and the accuracy of the data analysis; contributed substantially to the study design (analysis plan), data analysis, reporting, interpretation, and the writing of the manuscript.

## FUNDING INFORMATION

Actelion Pharmaceuticals US, Inc. sponsored this study. IQVIA received funding from Actelion Pharmaceuticals US, Inc. for the execution of the study.

## NOTATION OF PRIOR ABSTRACT PUBLICATION/PRESENTATION

Yeaw J, Lickert C, Drake W, De AP, Wade RL. Treatment Patterns among Patients Prescribed PDE-5 Inhibitor monotherapy For Pulmonary Arterial Hypertension. International Society for Pharmacoeconomics and Outcomes Research (ISPOR) 21st Annual International Meeting, Washington D.C. May 21-25, 2016.

## ACKNOWLEDGMENTS

Carole Alison Chrvala, PhD of Health Matters, Inc., funded by Actelion Pharmaceuticals US, is acknowledged for her assistance with the development of this manuscript based on early conversations with the authors. Donna Simcoe, MS, MBA, CMPP, of Simcoe Consultants, Inc. is acknowledged for her oversight of the manuscript development process and provision of editorial support.
